# Helium–Oxygen Mixture Model for Particle Transport in CT-Based Upper Airways

**DOI:** 10.3390/ijerph17103574

**Published:** 2020-05-20

**Authors:** Mohammad S. Islam, YuanTong Gu, Arpad Farkas, Gunther Paul, Suvash C. Saha

**Affiliations:** 1 School of Mechanical and Mechatronic Engineering, University of Technology Sydney (UTS), 15 Broadway, Ultimo, NSW 2007, Australia; mohammadsaidul.islam@uts.edu.au; 2 School of Mechanical, Medical and Process Engineering, Queensland University of Technology, Brisbane, QLD 4001, Australia; yuantong.gu@qut.edu.au; 3 Centre for Energy Research, Konkoly-Thege M. Street 29-33, 1121 Budapest, Hungary; farkas.arpad@energia.mta.hu; 4 Australian Institute of Tropical Health and Medicine, James Cook University, Townsville, QLD 4810, Australia; gunther.paul@jcu.edu.au

**Keywords:** targeted drug delivery, helium–oxygen mixture, particle transport, particle deposition, mouth–throat model

## Abstract

The knowledge of respiratory particle transport in the extra-thoracic pathways is essential for the estimation of lung health-risk and optimization of targeted drug delivery. The published literature reports that a significant fraction of the inhaled aerosol particles are deposited in the upper airways, and available inhalers can deliver only a small amount of drug particles to the deeper airways. To improve the targeted drug delivery efficiency to the lungs, it is important to reduce the drug particle deposition in the upper airways. This study aims to minimize the unwanted aerosol particle deposition in the upper airways by employing a gas mixture model for the aerosol particle transport within the upper airways. A helium–oxygen (heliox) mixture (80% helium and 20% oxygen) model is developed for the airflow and particle transport as the heliox mixture is less dense than air. The mouth–throat and upper airway geometry are extracted from CT-scan images. Finite volume based ANSYS Fluent (19.2) solver is used to simulate the airflow and particle transport in the upper airways. Tecplot software and MATLAB code are employed for the airflow and particle post-processing. The simulation results show that turbulence intensity for heliox breathing is lower than in the case of air-breathing. The less turbulent heliox breathing eventually reduces the deposition efficiency (DE) at the upper airways than the air-breathing. The present study, along with additional patient-specific investigation, could improve the understanding of particle transport in upper airways, which may also increase the efficiency of aerosol drug delivery.

## 1. Introduction

Our general understanding of the airflow and inhaled particle transport in the human lung is improved by a wide range of numerical studies published in literature [1,2,3,4]. The development of anatomically more and more realistic models and the availability of high-performance computing facilities help the analysis of the mechanisms and effects implicated in particulate matter transport and deposition in the respiratory tract. Administration of aerosol drugs through the oral route is preferred because of the lower filtering efficiency of the oral pathway compared to the nasal one resulting in a higher probability of drug penetration into the lungs as the targeted region [5,6,7]. However, several studies reported that even assuming oral inhalation, a significant fraction of the drug particles deposit at the mouth–throat and tracheal regions and do not reach the drug receptors. Although significant improvements were obtained in the last few decades, even the most efficient drug delivery devices cannot deliver more than 50–60% of the active ingredient to the lungs [8]. It is worth noting that additionally incorrect use of the device or the patient’s improper breathing pattern may further drastically reduce these values, thus necessitating an improved drug delivery efficiency. It is well-known that the highly-dense air influences flow patterns in the oral region and produces high turbulence intensity, especially at the level of the throat and glottis [9]. High levels of turbulence and fluctuation impact the overall deposition pattern at the oral airways and increase the drug aerosol deposition in unwanted areas. Inhalation of a less dense gas could potentially reduce turbulence and fluctuation at the oral airways, which may improve overall particle transport into the lower airways. A number of studies have investigated the transitional flow behavior at the upper airways and employed different turbulence models. Longest et al. [10] used laminar, and LRN k-ω model for airflow prediction at upper airways and found Reynold’s number is below the critical value of turbulence. However, this study did not consider oral-airways. A lattice-Boltzmann (LBM) based study analyzed the inhalation and exhalation for asymmetric tracheobronchial airways and found the Reynolds number is 1250, and this study did not consider the extra-thoracic airways [11]. A numerical analysis on the triple bifurcation model of the tracheobronchial airways used the LRN k-ω model and reported turbulence affects the airflow up to the first 5 generations at 30 lpm [12]. Another numerical study investigated the particle transport for the first 16 generations of a non-realistic model and used the LRN k-ω model [13]. This study did not analyze the turbulence behavior and reported that inertial and geometrical parameters affect the deposition pattern. An LBM approach comprehensively discussed the airflow and secondary vortex pattern in the upper airways [14]. This study analyzed the Womersley number effects on the flow structure in the upper airways. A computational study calculated the particle deposition for a large-scale non-realistic model and analyzed the computational cost for the multigenerational model [15]. This study did not discuss any turbulence effects on airflow in the airways. Feng et al. [16] performed the particle transport in a triple-bifurcation model and reports that the experimental flow becomes turbulent for the Reynolds number range 283–4718. A comprehensive LBM based study investigated the particle deposition in the upper airways and reports larger particle inertia influence the microparticle deposition in the upper airways [17]. All of these studies did not consider the oral airways and a range of studies reported that flow become locally turbulent at the extra-thoracic and upper airways for fluid flow ≥30 lpm [9,18,19].

The compound mixture of helium and oxygen with typical ratios of 80:20 or 70:30 is known as heliox. By its physical nature, it may benefit in minimizing the turbulence and fluctuation at the oral airways. The breathing gas helium is non-toxic and less dense than other gases, and this property makes helium popular in respiratory therapeutic and clinical applications. A heliox mixture of 80:20 ratio is 2.5 times less dense than air, and the non-toxic gas mixture minimizes the pressure gradient at the oral airways as turbulence and fluctuation are reduced for low-density gases. Helium gas was first used for asthma patients in 1935 [20], and heliox inhalation therapy is widely used in different clinical applications for acute asthma [21], chronic obstructive pulmonary disease [22,23], and bronchiolitis patients [24]. While experimental and clinical heliox therapy studies have improved the understanding of airflow and airway resistance under respiratory disease conditions, the mechanism of drug aerosol transport to the targeted airways is still not agreed in the available literature. Only a few computational studies have addressed flow and aerosol transport in human lung when inhaling heliox. A numerical approach used a heliox mixture model and reported a low-pressure gradient for the heliox mixture model compared to air [25]. Recently, a computational study employed a heliox mixture for airflow and aerosol transport analysis in a CT-based airway model and predicted the velocity flow field for different breathing conditions [26]. This study reported that the DE using a heliox mixture model was 2.2 times lower compared to air-breathing. Conversely, the above computational study did not consider the mouth–throat region in its overall calculations. It is, however, important to consider the oral airways for a better understanding of aerosol transport to the tracheobronchial and terminal airways. The primary aim of this study is to provide a detailed analysis of the implications of heliox inhalation concerning the upper airway deposition by using a CT-based upper and large bronchial airway model. 

## 2. Airway Geometry, Numerical Grid Refinement, and Validation

**Figure 1:**
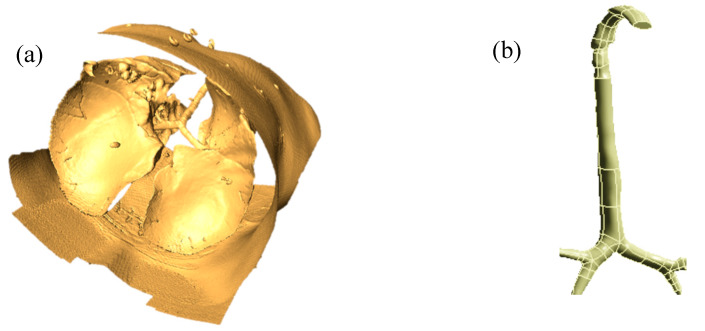
Three-dimensional (3D) anatomical model construction from the CT-images; (**a**) raw anatomical model of the whole lung, and (**b**) 3D upper and large bronchial airway model.

The airway model was reconstructed from DiCom images of CT-scan data. Medical images of one 55-year-old adult subject were used for the overall procedure, and different geometry generation software, AMIRA and Geomagic, were applied to image-processing. Details of the segmentation and reconstruction procedures can be found in the authors’ previous work [27]. Figure 1a shows a raw 3D model of the whole lung, and Figure 1b shows a processed digital replica of the upper and large bronchial airways considered in this study. 

**Figure 2:**
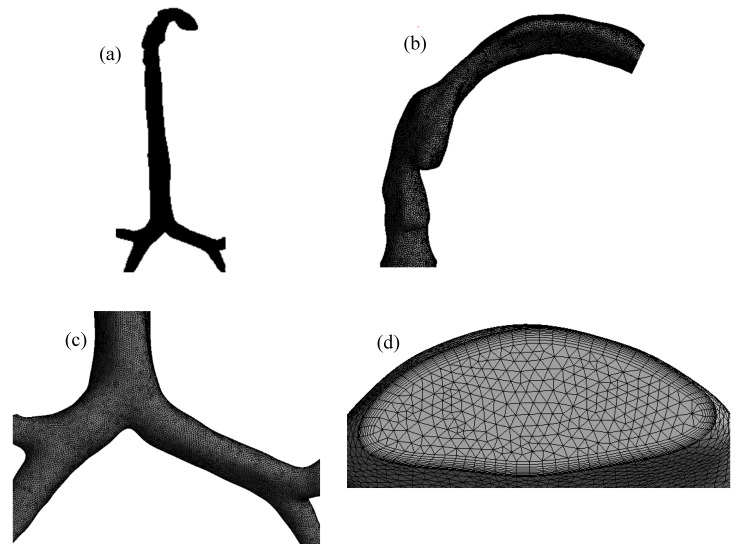
Unstructured mesh for the considered airway model; (**a**) mesh for the entire model (invisible due to a large number of small cells), (**b**) tetrahedral elements for the mouth–throat section, (**c**) surface mesh of the tracheobronchial airways, and (**d**) inflation mesh at the inlet section of the trachea.

**Figure 3:**
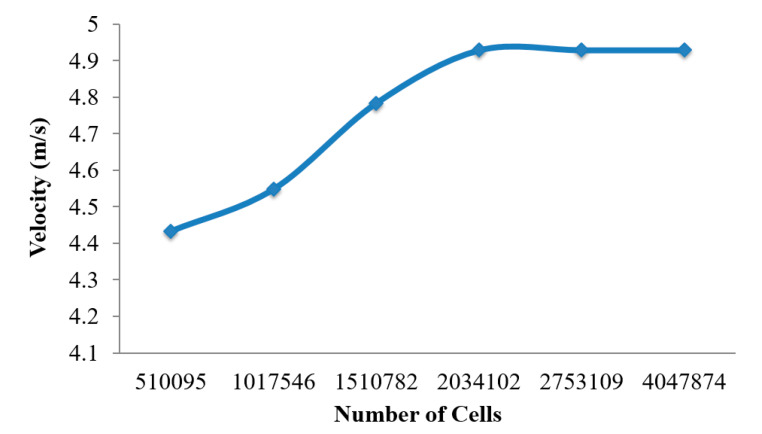
Mesh independence test results for a different number of cells.

**Figure 4:**
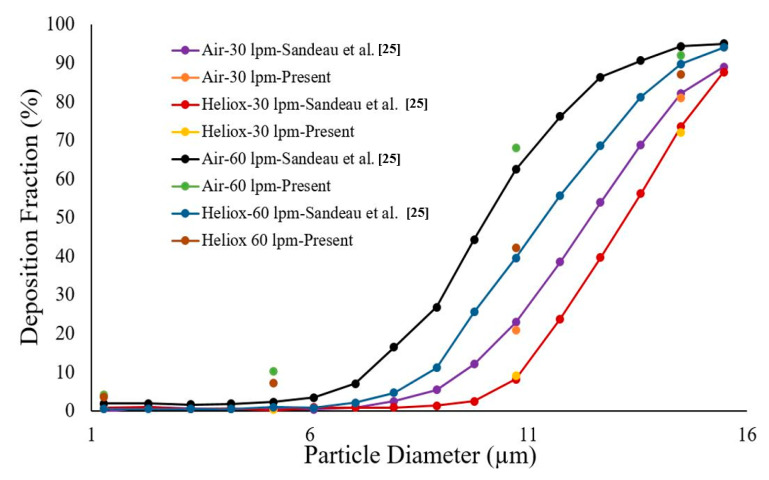
Deposition fraction comparison for various inlet flows with available literature.

The mesh of the highly asymmetric mouth–throat and large bronchial airway model (Figure 2) consists of unstructured tetrahedral elements. ANSYS meshing-module was employed to create the mesh for this airway model. An inflation layer mesh was generated for the mouth–throat and bronchial airway wall. The inflation layer consists of hexahedral elements, which can better capture the salient features of the flow near the wall boundary. Grid independence was confirmed (Figure 3), and the final mesh contained 2.75 million mixed cells. In all, 5.96 million mixed interior faces were generated for the whole model. The inflation layer consists of 10 prismatic layers near the wall with smaller cells approaching the wall, and the cell-to-cell growth rate was 1.1 when nearing the core region. The Y+ value of the final mesh is ≤1. A comprehensive validation for micro-particle transport at the mouth–throat and upper airways was validated at the authors’ previous study [28] (Figure 4). The particle transport and corresponding deposition fraction at the extrathoracic airway for heliox breathing were compared with the available data, and Figure 3 shows the present heliox model showing a good match with all cases of the previous investigation. The deposition fraction for the 60 lpm case during air-breathing shows a deviation with the previous investigation. For the 5-µm diameter particle, the present study indicates about 7.5% error, and about 5% error for the 10-µm diameter particle. However, the 15-µm particle is in line with the previous data. The calculated R^2^ value of the deposition fraction of the 60 lpm air case of the available literature is 0.9131, and the R^2^ value for the present 60 lpm air inhalation is 0.964. 

## 3. Numerical Methods

Navier–Stokes equations expressing the conservation of mass and momentum were solved to simulate the flow of gas.

(1) $$\frac{\partial \rho }{\partial t}+\nabla .\left(\rho \vec{v}\right)=0$$

*ρ* is the density of the continuum phase, and *t* is the time.

(2) $$\frac{\partial }{\partial t}\left(\rho \vec{v}\right)+\nabla \cdot \left(\rho \vec{v}\vec{v}\right)=-\nabla p+\nabla \cdot \left(\mu \left[\left(\nabla \vec{v}+\nabla {\vec{v}}^{T}\right)-\frac{2}{3}\nabla \cdot \vec{v}I\right]\right)+\rho \vec{g}+\vec{F}$$

The gravitational body force is $\rho \vec{g}$, and $\vec{F}$ is the interaction body force. $\vec{v}$ is the velocity vector field. Molecular viscosity is defined as *µ* and the unit tensor is *I*. 

Fluid and particle were considered as continuous and disperse phases, respectively. The Lagrangian approach was employed for aerosol transport prediction. The particle transport governing equation was solved for aerosol transport at the upper airways. Newton’s second law was used to calculate particle motion.

(3) $$m_{p,i}\frac{\partial {\vec{v}}_{p,i}}{\partial t}={\vec{F}}_{D,i}+m_{p,i}\vec{g}$$

(4) $$F_{D}\;\mathrm{is}\;\mathrm{the}\;\mathrm{drag}\;\mathrm{force}\;\mathrm{and}\;{\vec{F}}_{D,i}=\frac{1}{2}C_{D}\frac{\pi d_{p,i}^{2}}{4}\rho \left({\vec{v}}_{p,i}-\vec{v}\right)\left|{\vec{v}}_{p,i}-\vec{v}\right|$$

where, $m_{p,i}$ is the mass of the disperse phase, $C_{D}$ is the drag coefficient. Continuous phase (fluid) and discrete phase one-way coupling was considered in this study. The energy equation and species transport model were used to model the helium–oxygen gas mixture. The species transport equation for the *i*th species is

(5) $$\frac{\partial }{\partial t}\left(\rho Y_{i}\right)+\nabla .\left(\rho \vec{v}Y_{i}\right)=-\nabla .{\vec{J}}_{i}+R_{i}+S_{i}$$

where, $Y_{i}$ is each species local mass fraction. ${\vec{J}}_{i}$ is the *i*th species diffusion flux and can be defined as

(6) $${\vec{J}}_{i}=-\left(\rho D_{i,m}+\frac{\mu_{t}}{S_{C_{t}}}\right)\nabla Y_{i}-D_{T,i}\frac{\nabla T}{T}$$

where $D_{i,m}$ is the species diffusion coefficient, $\mu_{t}$ is turbulent viscosity, turbulent Schmidt number is *S_Ct_*. The turbulent Schmidt number is constant 0.7. *D_T_* is turbulent diffusivity. 

*R_i_* is the species production rate, and it can be defined as

(7) $$R_{i}=M_{w,i}\overset{N_{R}}{\underset{r=1}{\sum }}\hat{R_{i,r}}$$

*M_w,i_* is molecular weight and $\hat{R}$*_i,r_* is molar creation rate of species. *S_i_* is the rate of creation by addition from the dispersed phase, which is zero for this study. 

Inlet diffusion and diffusion energy source options were used in this study. A helium–oxygen-mixture template with 80% helium and 20% oxygen was created for the mixture material. The incompressible-ideal gas method for density and the mixing law for specific heat were used. The constant-dilute-appx method was used to model the mass diffusivity of the species. The properties of helium and oxygen are presented in Table 1.

The standard k-ω turbulent model was used as the viscous turbulence model, and the detail about the model can be found in the Supplementary Section. The calculated maximum Reynolds number of this study was 6956. Different inlet velocities, corresponding to inlet flow rates of 30 and 60 L/min, were used for the calculations. A zero gauge pressure condition was employed at the outlets; however, a minor pressure at the terminal airways is possible. This species transport study considered up to the first three bifurcations, and an open outlet condition was used. Inert particles with a density of 1100 kg/m^3^ [29] were released from the inlet of the mouth–throat. The particles were introduced at once, and the rosin-rammler method [29] was used to simulate polydisperse particle distribution. The flow rate was scaled by using the face area of the mouth–throat. Particle injection refinement on deposition was performed, and a total of 117,000 particles were released from the inlet. Non-uniform size distribution and spherical particles were used in the study. The mean diameter of the injection is 5e^−06^ µm. A “trap” wall condition was used for the airway wall [28]. The “trap” condition means the coefficient of restitution is zero, and when the particle touches the wall, there will be no bounce. The airway wall is stationary, and due to no-slip condition, the particle will stick at the wall. The particle diameter and corresponding Stokes numbers are listed in Table 2. 

A fluent user-defined function (UDF) was employed to track the deposited particle. The trapped particle information was collected from the UDF. Tecplot post-processing tool and MATLAB code were used for deposition data analysis. The trapped particle concentration on the airway wall was calculated using the MATLAB code. A high-performance computational facility was used, and a single simulation took about 250 hours. Sixteen processors and 8 GPGPU per machine were used for the numerical calculation. The scaled residual convergence of the continuity and momentum equations was 0.0001. After the convergence of the steady airflow equation, particles were injected as a transient process. 

## 4. Results and Discussion

Airflow and particle transport through the upper and bronchial airways were simulated for both heliox and air-breathing conditions. Polydisperse aerosol particles were considered for the particle transport study as both atmospheric and drug delivery device-generated particles are polydisperse. 

**Figure 5:**
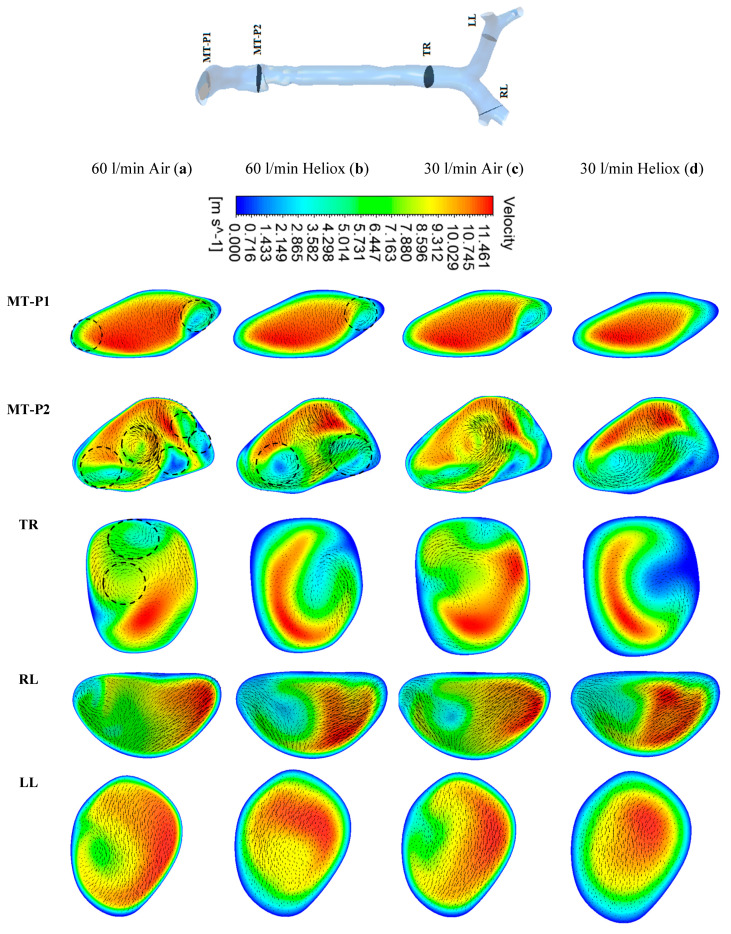
Velocity contours at selected positions of the upper airway at t = 1 s, (**a**) air—60 L/min, (**b**) heliox—60 L/min, (**c**) air—30 L/min, and (**d**) heliox—30 L/min. MT-P1, mouth–throat plane 1; MT-P2, mouth–throat plane 2; TR, trachea; RL, right lung; LL, left lung.

**Figure 6:**
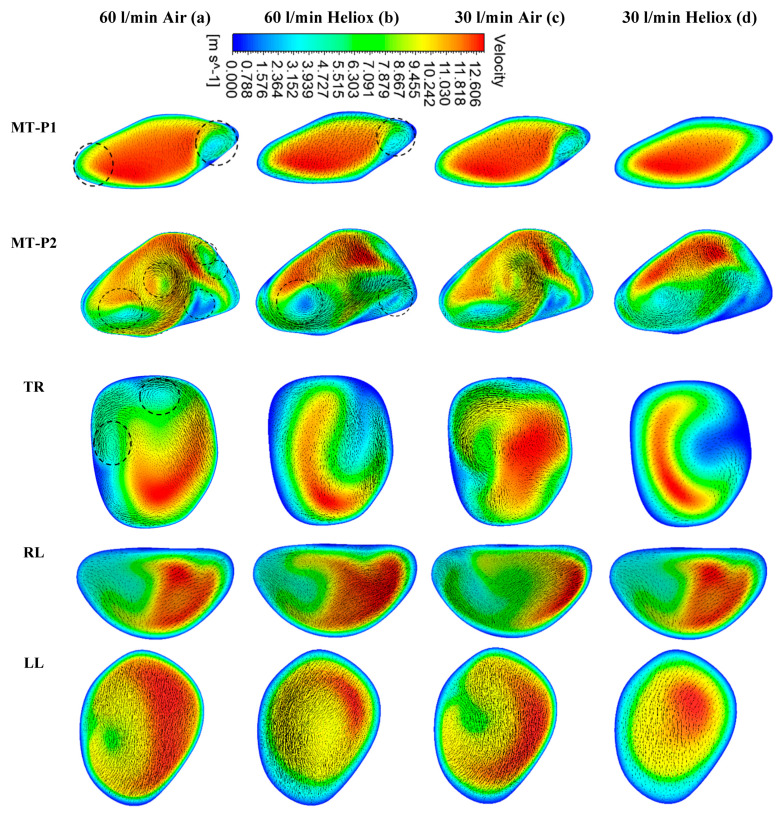
Velocity contours at selected positions of the upper airway at t = 2 s, (**a**) air—60 L/min, (**b**) heliox—60 L/min, (**c**) air—30 L/min, and (**d**) heliox—30 L/min. MT-P1, mouth–throat plane 1, MT-P2, mouth–throat plane 2, TR, trachea, RL, right lung, LL, left lung.

Figure 5 and Figure 6 present the simulated velocity contours at selected positions of the mouth–throat and bronchial airways at t = 1 s and t = 2 s, respectively. The study considered heliox and air-breathing. Figure 5a,b depicts the velocity contours at 60 L/min flow rate for air and heliox breathing. At t = 1 s, the velocity contours at the mouth–throat region (MT-P1 and MT-P2) show a more complex flow field for air-breathing than for helium–oxygen mixture breathing. At MT-P1, air-breathing resulted in two vortices, while the heliox breathing shows only one vortex solution. At MT-P2, the flow field for air inhalation shows a highly complex velocity pattern with a five vortex solution. On the contrary, the helium–oxygen mixture breathing shows a two-vortex solution. The velocity contour at the tracheal section also shows a similar flow field for air inhalation. The velocity vectors of the corresponding velocity contours illustrate the highly complex flow field at the mouth–throat and tracheal area. The overall velocity contour and velocity vector for 60 L/min flow rate illustrate that flow becomes locally complex at the oral airway for air inhalation. The highly dense air yields a more complex flow field than a heliox mixture. At 30 L/min flow rate, air inhalation also shows a similar flow field at the oral and tracheal wall as at 60 L/min flow rate. At t = 2 s, the velocity contour shows a similar vortex pattern as at t = 1 s for air and heliox breathing (Figure 6). However, at t = 2 s, the velocity magnitude at the selected position of the airways was found slightly higher than t = 1 s. 

**Figure 7:**
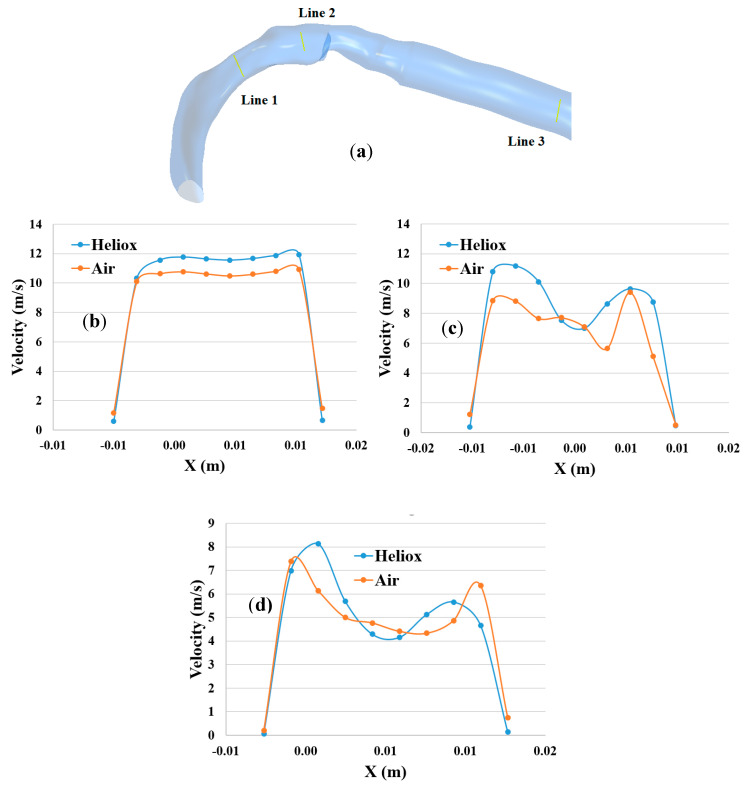
Velocity profile at selected positions of the airways at 60 lpm, (**a**) selected line at oral airway, (**b**) line 1, (**c**) line 2, and (**d**) line 3.

The overall velocity and velocity vector contours for air-breathing are found significantly other than in the case of heliox inhalation. The velocity contours for heliox breathing show a lower velocity near the airway wall, while air-breathing shows higher velocity near the airway wall. Velocity profiles were plotted at the selected positions of the mouth–throat and trachea for a better understanding of the airflow pattern. Figure 7 shows the velocity profiles for air and heliox at the 60 lpm case. Figure 7b shows the velocity profile at the beginning of the mouth–throat area, and the velocity magnitude at the middle of the mouth–throat wall is higher for heliox than the air-breathing. However, the near-wall velocity for air is found higher than the heliox. Figure 7c shows the velocity profile in the middle of the mouth–throat model (line 2), and the velocity profile shows an intricate flow pattern at this position. The velocity profile for air at line 2 is found more complex than the heliox. A similar velocity flow filed is observed in the tracheal region of the airway, which is shown in Figure 7d. The highly complex structure of the oral airways and high flow rates influence the flow pattern in the upper airways.

**Figure 8:**
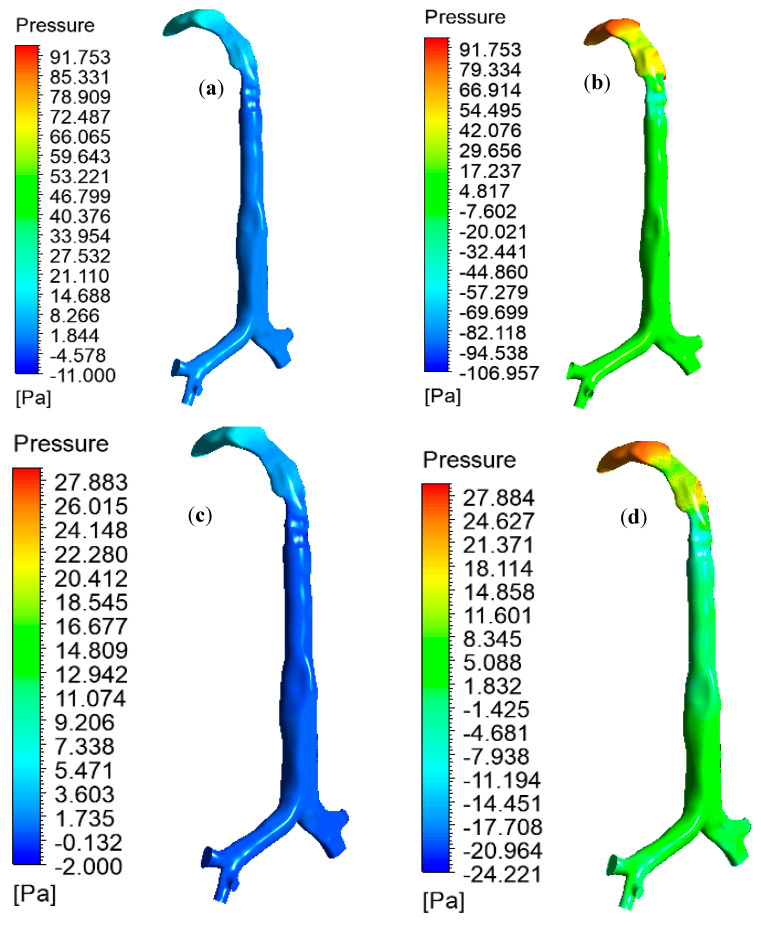
Pressure variation in the mouth–throat and upper airways for various inlet conditions, (**a**) heliox—60 L/min, (**b**) air—60 L/min, (**c**) heliox—30 L/min, and (**d**) air—30 L/min (same pressure scale is used for comparison purpose).

The pulmonary pressure variations at the mouth–throat and large bronchial airways play an important role in the mechanical ventilation of the airways during different disease conditions. The high-pressure variation across the oral and upper airways indicates a high resistance and stress on the airway, which could lead to ventilator-induced airway injury [30]. A proper understanding of pressure variation at the upper airways is essential for managing the breathing procedure of asthma and COPD patients. Figure 8 reports the pressure drop at the oral and large bronchial airways for various inlet conditions. During heavy breathing (60 L/min), heliox breathing pressure resistance is 4.68 times less at the oral and large bronchial airways than for air-breathing. Pressure profiles show less pressure at the upper airways during heliox inhalation than for air inhalation. This particular finding could potentially help the respiratory treatment of asthma and COPD patients. A lower pressure resistance at the oral airways will particularly help with the mechanical ventilation of aged asthma and COPD patients. For the lower 30 L/min flow rate, heliox inhalation also shows less pressure than air inhalation. The pressure at different position of the lung are calculated, and Table 3 shows the pressure magnitude at selected planes of the lung airway. 

**Figure 9:**
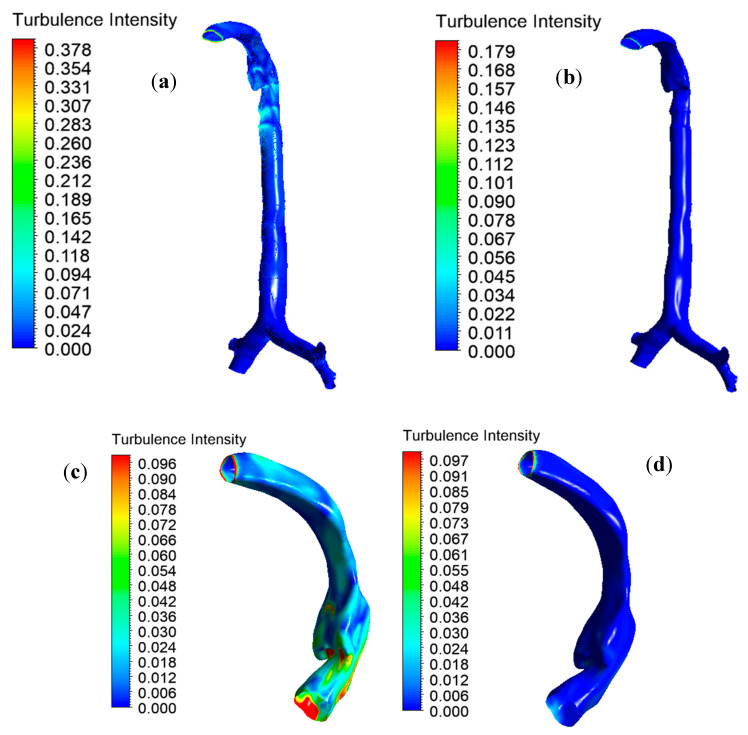
Turbulence intensity for different gas inhalation conditions at 60 L/min flow rate, (**a**) air inhalation upper airway, (**b**) heliox inhalation upper airway, (**c**) air inhalation mouth–throat region, and (**d**) heliox inhalation mouth–throat region.

Knowledge of the airflow dynamics in the oral and tracheobronchial airways is important for improved analysis of disease conditions and for more efficient drug delivery. The available literature reports that the flow pattern becomes turbulent in the oral airways during inhalation at moderate to heavy exercise conditions (Q > 30 L/min) [9,31]. In the local mouth–throat and tracheal areas, turbulent fluctuation affects drug-aerosol transport and increases unwanted deposition. Turbulent dispersion at the mouth–throat and large bronchial airways result from turbulence intensity profiles for the various inlet conditions, as shown in Figure 9. Turbulence intensity is the ratio of the root mean square of the velocity fluctuation to the mean flow velocity. Figure 9a,b reports a 2.11-times lower maximal turbulence intensity for the heliox mixture than for air inhalation. The lower density of the heliox mixture minimizes overall turbulence intensity at the oral airways. Figure 6c,d shows the intensity profile scaled to a customized range for better visualization. The turbulence profile shows the higher turbulence intensity in the oral airways for air inhalation. The quantitative value of turbulence intensity at different positions of the lung is presented in Table 4. 

**Figure 10:**
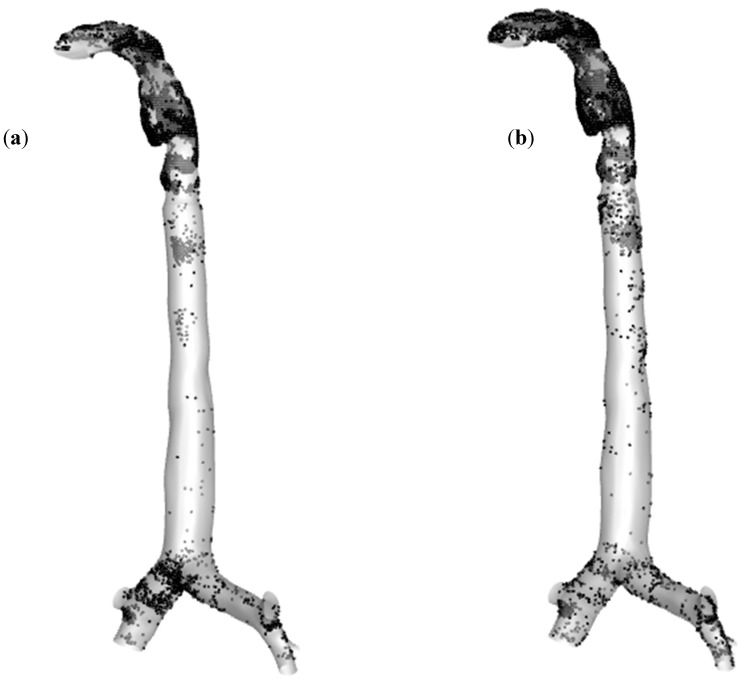
Aerosol deposition scenario for 60 L/min flow rate, (**a**) heliox breathing, and (**b**) air breathing.

Figure 10a,b shows the polydisperse particle deposition patterns in the oral and upper airways at 60 L/min flow rate for air and heliox inhalation. In Figure 10b, it can be seen that a larger amount of particles are deposited at the mouth–throat area and large bronchial airways when breathing air, compared to breathing heliox in Figure 10a. The highly complex anatomical shape of the mouth–throat, higher pressure resistance, and turbulence intensity influence the deposition pattern when inhaling air. 

**Figure 11:**
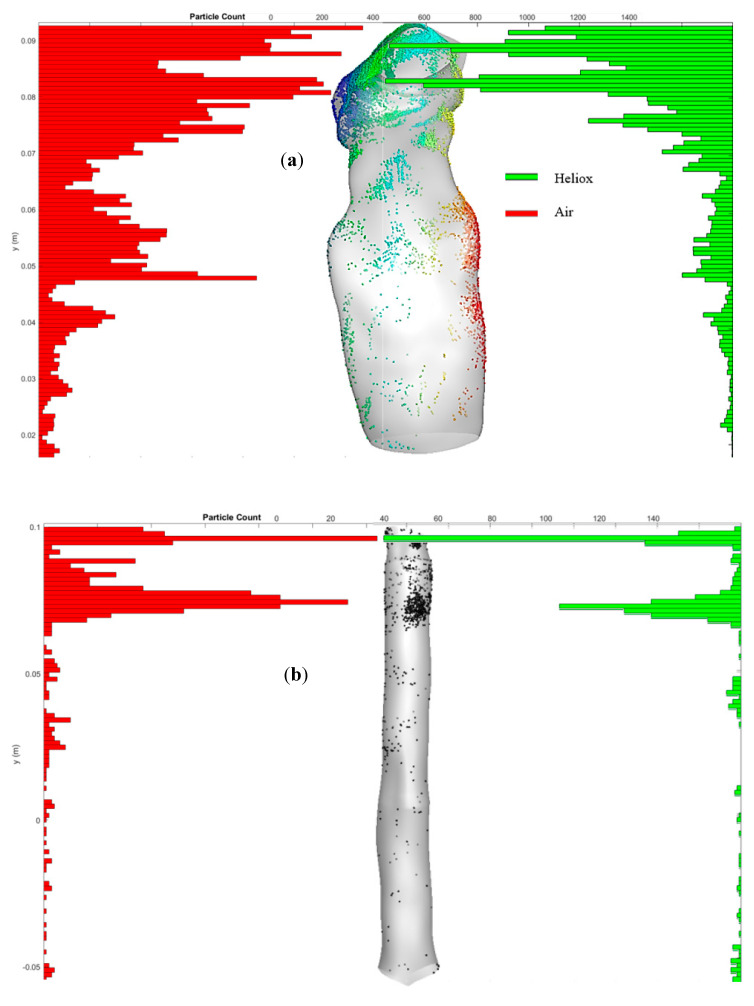
Particle deposition concentrations for air and heliox, (**a**) mouth–throat region; particles are counted based on a reference y-position in the mouth–throat, and (**b**) tracheal region. Red (dark) bars represent air inhalation, and green (light) bars represent heliox inhalation.

To further study polydisperse micron size particle deposition along the airways, the particle deposition distribution is shown in Figure 11. Figure 11a reports the highly concentrated deposition zones of the mouth–throat airway for air and heliox inhalation. The distribution chart illustrates that a significant amount of particles are deposited at the upper and middle section of the mouth–throat for air inhalation. For the heliox mixture, the chart reports noticeably lower particle concentrations throughout the mouth–throat model. The lower density of the heliox mixture reduces the particle deposition in the mouth–throat model. Similarly, Figure 11b shows the particle concentration at the tracheal wall area for heliox and air inhalation. Particles are mostly trapped at the upper portion of the tracheal wall, where the turbulence intensity is found highest. 

**Figure 12:**
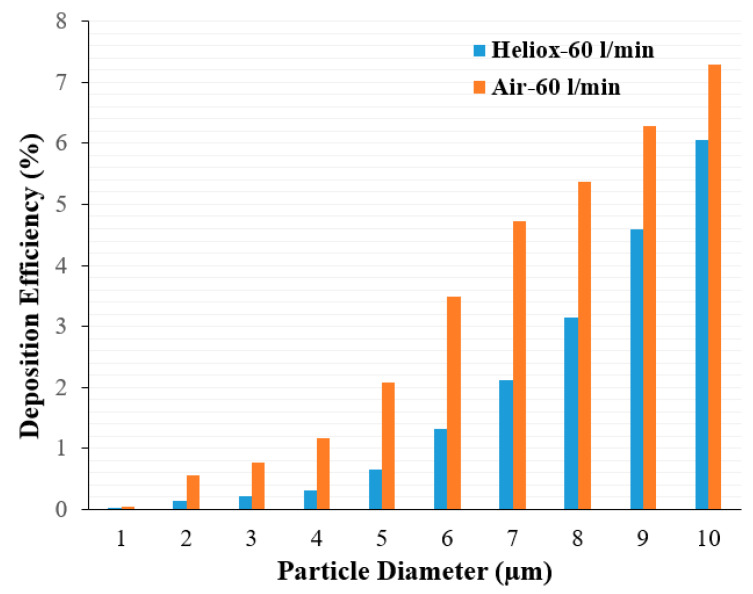
Polydisperse particle desposition effciency (DE) comparison in the mouth–throat area at 60 L/min.

Analysis of the polydisperse particle DE at the mouth–throat area provides further insight. Figure 12 shows DE for air and heliox inhalation at 60 L/min flow rate. DE is always higher for air inhalation and shows an increasing trend with particle size. For the microparticle, inertia plays an important role. At a higher inhalation rate, micro-diameter particles deviate from the air trajectory due to their higher inertia when the particle faces any irregular pathway. The mouth–throat shape is highly irregular, which influences turbulence intensity at 60 L/min flow rate. Air flow rate, turbulence intensity, complex irregular shape, and larger inertia all significantly increase DE at the mouth–throat area. For a heliox gas mixture, the flow becomes less turbulent due to a lesser gas density, which then decreases the overall DE at the mouth–throat area.

**Figure 13:**
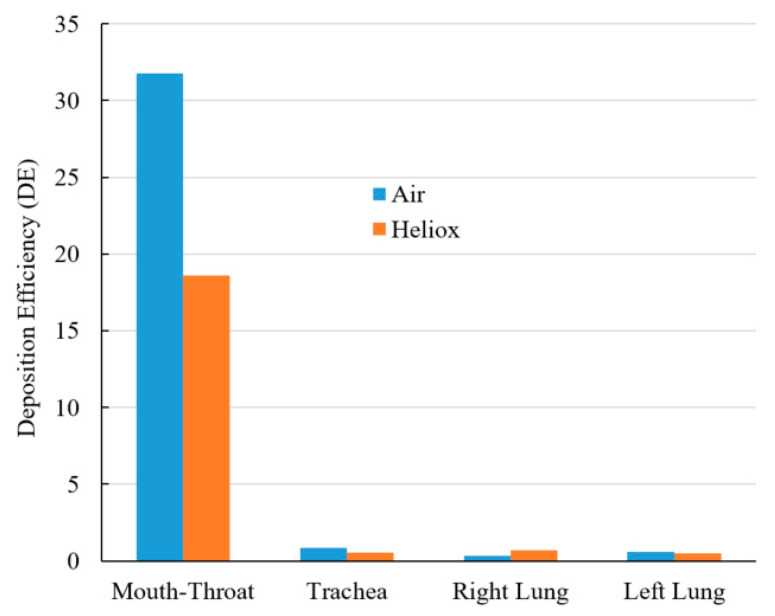
DE of air and heliox inhalation at 60 L/min flow rate at various airway positions.

Figure 13 shows a DE comparison for air and heliox inhalation. The chart shows that particles are mostly trapped at the oral airways. For air-breathing, more than 31% of particles are deposited at the mouth–throat region, which is 1.71 times higher than the deposition rate for helium-oxygen gas inhalation. The numerical results demonstrate that the less dense heliox mixture significantly decreases aerosol particle deposition at the oral airways, which is the key finding of this study. As a result of the lower deposition rate in the oral airways, more particles will be transported towards the lungs. DE at the tracheal wall was also found lower for heliox breathing, essentially supporting the key finding of this study. While DE when inhaling heliox was found lower in the left lung, it was found higher in the right lung. 

**Figure 14:**
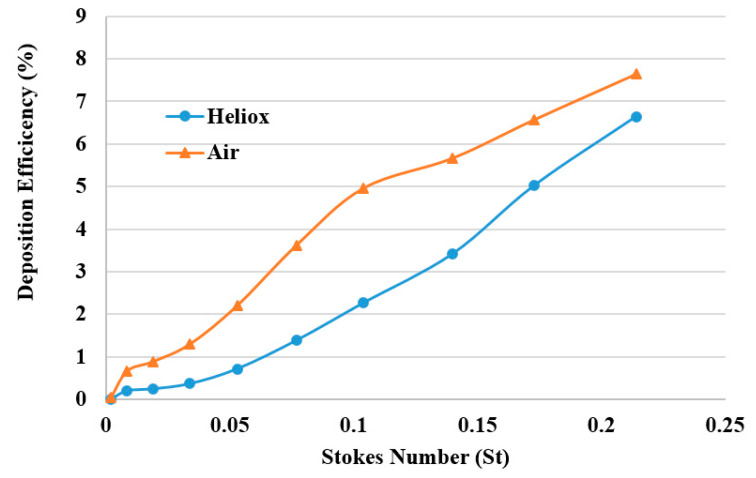
DE comparison against Stokes number for various breathing methods.

The DE was calculated against Stokes number St = ρd_p_^2^U/18µD for different breathing methods (Figure 14). The overall DE curve for both heliox and air-breathing shows an increasing trend with the Stokes number, which aligns with the general deposition pattern of the microparticle. The higher Stokes number shows higher DE, which indicates that microparticle inertia and inertial impaction influence the overall deposition pattern. 

**Figure 15:**
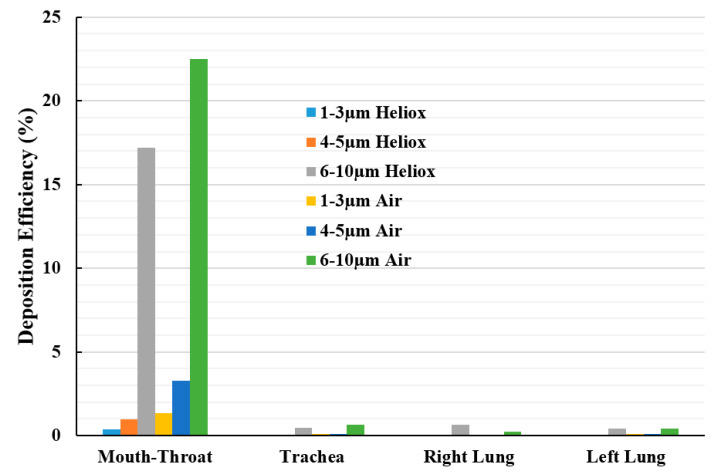
Deposition hot spot comparison for different diameter particles at 60 lpm inlet conditions.

Figure 15 reports the deposition hot spot for different diameter particles during heliox and air inhalation. The DE calculations were carried out for three ranges of particle diameter. The overall investigation reports particles ranging from 6–10 µm diameter were mainly deposited at the mouth–throat area of the lung. The DE of smaller cluster particles (1–3 µm) at the mouth–throat and upper airways was found relatively low, and most of these particles are escaped through the outlets of the airways. 

## 5. Summary and Perspectives

In summary, a modeling framework for air and a helium–oxygen gas mixture was developed to predict the airflow and aerosol transport at the mouth–throat and upper airways. Different inlet conditions were considered for the airflow to predict aerosol transport. A realistic airway model was employed for the numerical calculation. The key outcomes of this study are

- The velocity field of inhaled air is more complex than the velocity field of heliox inhaled at the same flow rate. For instance, at 60 L/min, a five vortex solution is observed for air, while heliox shows a two vortex solution;
- Pressure resistance varies significantly for different inlet conditions. Air inhalation results in about 4.7 times higher pressure than a heliox mixture;
- The turbulence intensity for air inhalation is about 2.1 times higher than for heliox. High turbulence intensity influences the deposition in the oral airways;
- The heliox mixture model reduces the overall DE, especially in the mouth–throat and, to some extent, also in the trachea and large bronchi. For air inhalation, about 34% of the injected particles are trapped, compared to about 20% for heliox inhalation.

The outcome of this study warrants more case studies to improve the understanding of this problem. Pressure variation along the airway for heliox inhalation is observed, which would potentially help the treatment of asthma and COPD patients. Turbulence intensity and DE predictions will help to improve drug delivery to the lower airways. Future studies should consider patient-based airflow analysis and analysis of aerosol deposition in the lower airways. Some future recommendations are listed below;

- This study used a constant inlet velocity. In real life, inlet velocity varies from person to person, and the velocity profile at the inlet is highly complex.
- This study used the k-ω turbulence model for airflow characterization. However, for transitional flow, large eddy simulation (LES) can more accurately predict the transitional behavior of the flow.
- Airway deformation was not considered for this study.

## Figures and Tables

**Table 1:** Properties of oxygen and helium.

	Oxygen	Helium
Density (kg/m^3^)	1.299	0.1625
Viscosity (kg/m-s)	1.919 × 10^−5^	1.910 × 10^−5^
Thermal Conductivity (w/m-k)	0.0246	0.152

**Table 2:** Particle information and Stokes number.

	Air Inhalation	Heliox Inhalation
Particle Diameter	1–10 µm	1–10 µm
Stokes number	0.0011–0.22	0.00107–0.2149

**Table 3:** Pressure (Pa) at selected planes in mouth–throat and upper airways.

	Air—60 lpm	Heliox—60 lpm	Air—30 lpm	Heliox—30 lpm
MTP1	78.77	17.07	24.47	5.44
MTP2	65.55	11.44	19.11	3.37
TR	13.28	2.95	7.65	0.82
RR	6.28	1.46	1.61	0.52
LL	3.87	1.19	1.35	0.32

**Table 4:** Turbulence intensity at different positions of the lung.

	Mouth–Throat	Trachea	Right Lung	Left Lung
60 lpm Air	0.3785	0.2574	0.0283	0.0659
60 lpm Heliox	0.179	0.017	0.0043	0.0043

## Supplementary Materials

The following are available online at https://www.mdpi.com/1660-4601/17/10/3574/s1, Section S1. Numerical Method; S2. Particle Distribution.
