# Supplementary material for: Helium–Oxygen Mixture Model for Particle Transport in CT-Based Upper Airways
Source: Int J Environ Res Public Health. 2020 May 20;17(10):3574. doi: 10.3390/ijerph17103574 (PMC7277378; doi:10.3390/ijerph17103574)
Supplement: Supplementary file 1 [file ijerph-17-03574-s001.pdf]

Helium–Oxygen Mixture Model for Particle Transport in CT-Based Upper Airways

Mohammad S. Islam [1], YuanTong Gu [2], Arpad Farkas [3], Gunther Paul [4] and Suvash C. Saha [1,*]

1 School of Mechanical and Mechatronic Engineering, University of Technology Sydney (UTS), 15 Broadway, Ultimo NSW-2007, Australia; mohammadsaidul.islam@uts.edu.au
2 School of Chemistry, Physics & Mechanical Engineering, Queensland University of Technology (QUT), 2 George Street, GPO Box 2434, Brisbane QLD 4001, Australia; yuantong.gu@qut.edu.au
3 Centre for Energy Research, Konkoly-Thege M. street 29-33, 1121 Budapest, Hungary; farkas.arpad@energia.mta.hu
4 James Cook University, Australian Institute of Tropical Health and Medicine, Townsville QLD 4810, Australia; gunther.paul@jcu.edu.au
* Correspondence: Suvash.Saha@uts.edu.au

S1. Numerical Method

Standard *k*-*ω* model is chosen based on the Wilcox [1] model, which incorporates modification of compressibility, low-Reynolds number effects and shear flow spreading. This model is also based on the the turbulence kinetic energy (*k*) and the specific dissipation rate (ω) transport equation.

The *k* and ω are obtained from the transport equations below:

$$\frac{\partial}{\partial t}(\rho k)+\frac{\partial}{\partial x_i}(\rho k u_i)=\frac{\partial}{\partial x_j}(\Gamma k\frac{\partial k}{\partial x_j})+G_k-Y_k+S_k \quad (1)$$

(1)

and

$$\frac{\partial}{\partial t}(\rho \omega)+\frac{\partial}{\partial x_i}(\rho \omega u_i)=\frac{\partial}{\partial x_j}(\Gamma \omega\frac{\partial \omega}{\partial x_j})+G_\omega-Y_\omega+S_\omega \quad (2)$$

(2)

where, Gk is the turbulence induced kinetic energy due to velocity gradients, Gω is the generation of ω . Effective diffusivity of k and ω are Γk and Γω respectively. Dissipation of k and ω are Yk and Yω respectively.

The diffusivities of the model are;

$$\Gamma_k=\mu+\frac{\mu_t}{\sigma_k}$$
$$\Gamma_\omega=\mu+\frac{\mu_t}{\sigma_\omega} \quad (3)$$

where turbulent Prandtl numbers are defined by σ_k and σ_ω. Turbulent viscosity is μ_t.

$$\mu_t=\alpha^*\frac{\rho k}{\omega}$$

Low- Reynolds correction is used for *k*-omega option. The coefficient α^* is given by

$$\alpha^*=\alpha_\infty^*(\frac{\alpha_0{}^*+\mathrm{Re}_t/R_k}{1+\mathrm{Re}_t/R_k}) \quad (4)$$

where $\mathrm{Re}_t=\frac{\rho k}{\mu\omega},\ R_k=6,\ \alpha_0{}^*=\frac{\beta_i}{3},\ \beta_i=0.072$ (5)

S2. Particle Distribution

Diesel exhaust particles are considered and the particle density 1,100 kg/m^3 is used. The spherical drag law is used for the particle. Previously known as the Weibull distribution, the Rosin and Rammler distribution (Rosin, 1933) is used to approximate the aerosol particle size distribution.

$$f(x;P_{80},m)=\begin{cases}1-e^{\ln(0.2)(\frac{x}{P_{80}})^m} & x\geq 0;\\ 0 & x<0,\end{cases} \quad (6)$$

where, x is the particle size, P_{80} is the 80th percentile of the particle size distribution, m is the spreading distribution parameter.

The inverse distribution is:

$$f(F;P_{80},m)=\begin{cases}P_{80}\sqrt[m]{\dfrac{\ln(1-F)}{\ln(0.2)}} & F>0;\\ 0 & F\leq 0,\end{cases} \quad (7)$$

Where F is the mass fraction.

The Rosin-Rammler distribution in Ansys (17.2) Fluent is the form of the Weibull distribution and requires the initial velocity, total flow rate, maximum, minimum and mean diameter, spread parameters. The spread parameter can be computed from the analytical equations

$$n=\frac{\ln(-\ln[Y_d])}{\ln\left(\dfrac{d}{\bar{d}}\right)} \quad (8)$$

where Y_d is the mass distribution function. The final Rosin-Rammler distribution function used is:

$$Y_d=e^{[-(\frac{d}{\bar{d}})^n]} \quad (9)$$

References

1. Wilcox, D.C., *Turbulence modeling for CFD*. DCW industries: La Cañada, CA, USA, 1998; Vol. 2.
